# Case Report: Improved Height in a Patient With Myhre Syndrome Using a Combination of Growth Hormone and Letrozole

**DOI:** 10.3389/fped.2021.675934

**Published:** 2021-07-29

**Authors:** Hui Wu, Xinli Wang, Yunpu Cui, Xuemei Wang

**Affiliations:** Department of Pediatrics, Peking University Health Science Center, Peking University Third Hospital, Beijing, China

**Keywords:** Myhre syndrome, growth hormone, letrozole, testicles, short stature

## Abstract

Myhre syndrome is a rare disorder caused by a heterozygous mutation in the *SMAD4* gene. Affected patients may exhibit dysmorphic facial features, intrauterine growth retardation, short stature, obesity, muscle hypertrophy, thickened skin, limited joint movement, hearing impairment, and varying degrees of psychomotor developmental disorder. Serious complications of the cardiovascular and respiratory system may be seen later in life. We report the case of a Chinese boy with Myhre syndrome presenting with a novel symptom of giant testicles where treatment with growth hormone combined with letrozole successfully improved his short stature. This case shows that letrozole combined with growth hormone can improve height in children with Myhre syndrome without adverse effects.

## Introduction

Myhre syndrome (MS, MIM 139210) is a rare autosomal, dominant, and hereditary disease caused by a mutation in the SMAD4 gene (1). The first case of MS was reported in 1981 (2). To date, 90 confirmed cases have been reported, with the youngest patient being 23 months old (3). Clinical manifestations include short stature, intrauterine growth restriction, dysmorphic facial features (blepharoptosis, prognathism, and small ears among others), varying degrees of cognitive impairment, decreased joint mobility, skin stiffness, and hearing impairment. Moreover, the common complications include hypertension, recurrent pericarditis, airway stenosis, and respiratory disorders. Few patients exhibit abnormal sexual development, such as premature puberty, cryptorchidism, secondary amenorrhea, and delayed sexual development (4). Additionally, skeletal abnormalities have been reported. Genetic testing has confirmed that almost all patients diagnosed with MS to date had de novo mutations in the SMAD4 gene, with only three cases resulting from an Arg496 residue mutation (5).

We report a case of a Chinese child with MS who was treated with growth hormone combined with letrozole and a yoga program.

## Case Description

The patient was the first child of healthy non-consanguineous parents, with no relevant family history. He was delivered by cesarean section for oligohydramnios at 38 weeks' gestation. His birth weight was 2,250 g (<P3), and his birth height was 45 cm (P3). Since birth, he had a slow growth rate and displayed difficulty squatting at age 3. At age 4 years and 9 months, he was 96 cm tall (<P3) and weighed 15 kg (P3); he was referred to a local hospital for growth retardation. His growth hormone level was normal, and no treatment was given at this time. At age 6, while his growth hormone level was still normal, he received growth hormone treatment for 1 year and his height increased by 10 cm; his parents then decided to stop the treatment.

**Figure 1:**
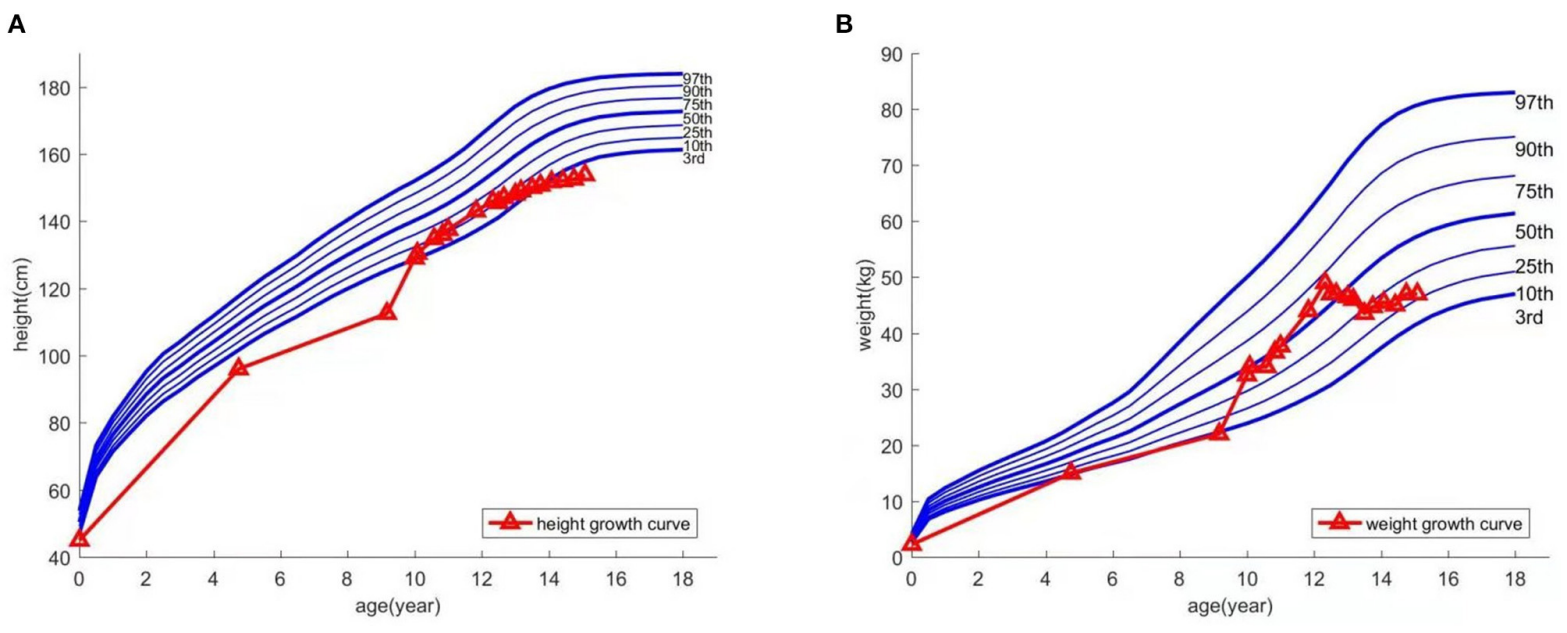
Growth curve of height **(A)** and weight **(B)**.

At age 9, the patient experienced an abnormally large testicular growth. Examination showed a testicular volume of 6 ml, Tanner stage of pubic hair stage III, and bone age of 12 years and 1 month. A gonadotropin-releasing hormone (GnRH) provocation test showed a peak luteinizing hormone (LH) of 12 mIU/ml, with no abnormalities observed on pituitary magnetic resonance imaging and adrenal ultrasound. He was diagnosed with central precocious puberty and received triptorelin for 8 months, which was then discontinued due to his increasing bone age. Furthermore, the patient resumed growth hormone treatment. During the course, hyperinsulinemia developed intermittently and was controlled after growth hormone withdrawal. The patient was hyperactive and inattentive from an early age; his Intelligence Quotient test score was 85. Integrated visual and auditory continuous performance tests indicated severe sensory integration disorder. Height and weight growth are shown in the growth curve (Figure 1).

**Figure 2:**
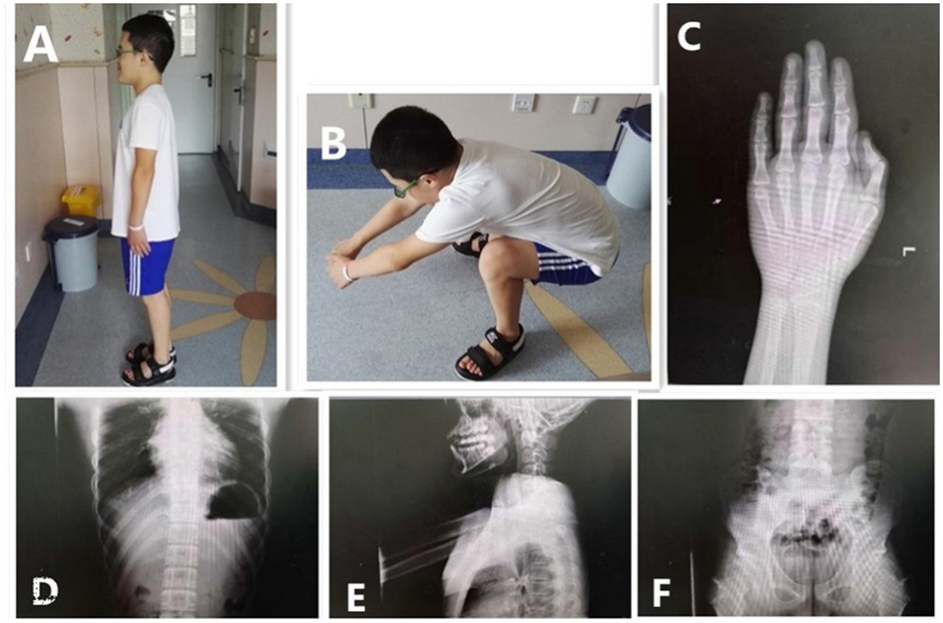
Clinical features and radiographs of the proband at 12.5 years. Special facial features: small ears, prognathism, and short fingers **(A)**. Difficulty in squatting **(B)**. The bone age in the image of the left hand was 16.5 years old; the phalanges were short **(C)**. Anteroposterior and lateral images of the spine showed wide ribs, flat vertebral bodies, large pedicles, and small and hypoplastic iliac wings **(D–F)**.

At age 12, he was referred to our outpatient clinic with the aim to further increase his height. His height and weight were 145.5 cm (P10) and 47 kg (P50-P75), respectively. Physical examination revealed abnormal facial features (short palpebral fissures, low nose bridge, small ears, short philtrum, prognathism) and a large body size. Short fingers, valgus elbows, and thick and rigid skin were observed (Figure 2). His testicular volume was 25–30 ml. His penis was 7 cm long, with a circumference of 9 cm. The Tanner stage of pubic hair was stage V.

Radiographic assessment indicated a bone age 16.5 years; his phalanges were short. No obvious abnormalities of the knees or spine were found; however, the vertebral bodies were flat with large pedicles. Wide ribs, hypoplastic iliac wings, and a small pelvis were noted. Echocardiogram, urinary ultrasound, and ultrasonography of the testicles and epididymis showed no abnormalities (Figure 2).

Laboratory tests revealed a slightly elevated glycosylated hemoglobin level A1c (HbA1c, 6.2%); the glucose tolerance and fasting insulin tests showed normal findings. Whole exome sequencing detected a heterozygous mutation in the SMAD4 gene (NM-005359.5, c.1498A > G).

Due to the wishes of the parents to increase the boy's height, and after full disclosure of the potential risks and uncertainties of treatment, growth hormone (7 IU/day), letrozole (2.5 mg/day), and metformin (250 mg/day, 3 times a day) were administered for 31 months. Over the treatment course, the boy's height increased by 9 cm. Blood pressure; thyroid, liver, and kidney function; glucose; fasting insulin; 25-hydroxyvitamin D3; HbA1c and blood lipid levels were normal. The LH and FSH levels slightly increased compared to the child's basal levels; however, they returned to normal 1 year later. Testosterone (T) increased transiently at treatment initiation and then decreased to normal. Following yoga, the child was able to squat normally.

**Figure 3:**
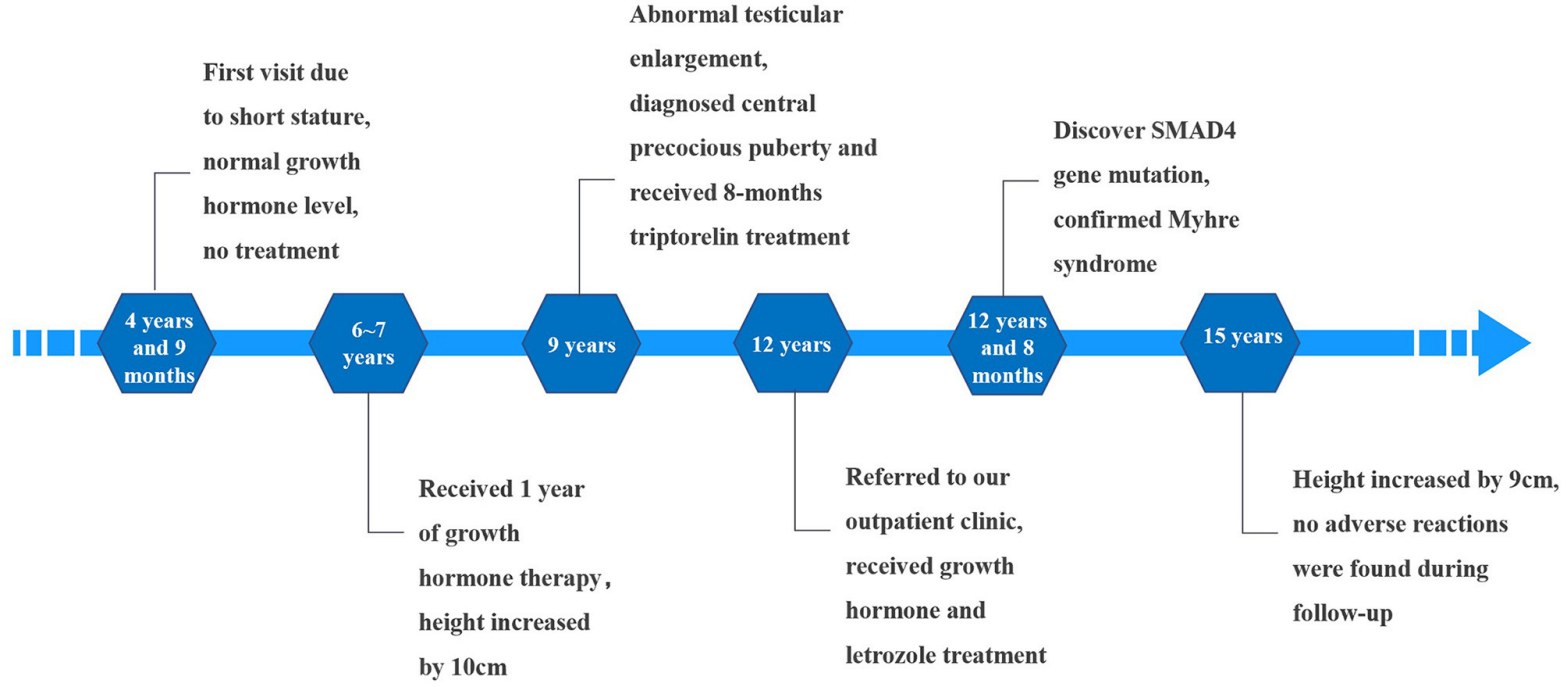
Timeline of the patient's treatment process.

The child was followed up until he was 15 years old, at which time he was 153.8 cm tall (<P3) and weighed 47 kg (P10–P25). There were no pulmonary or abdominal abnormalities observed during his last physical examination; his bone age had remained the same (16.5 years). Considering the side effects of long-term medication, the child's parents agreed to stop the treatment. Timeline of the patient's treatment process was showed in Figure 3. Informed consent and full permission for publication of this case report were obtained from the parents.

## Discussion

We reviewed more than 90 cases of patients with MS reported so far, and more than 80% had short stature. Unfortunately, short stature treatment was described in detail in few cases. Only seven cases were treated with growth hormone with diverse results, of which only three had good outcomes (6, 7). However, to the best of our knowledge, none have used growth hormone combined with letrozole treatment.

Letrozole is an aromatase inhibitor, which catalyzes the conversion of androgen to estrogen. Excessive estrogen can cause premature epiphyseal closure, and aromatase inhibitors may be used to delay epiphyseal closure, thereby increasing adult height. However, a main problem with aromatase inhibition is the possible adverse effects on bone mineralization, which was only described as transitory in children. Vertebral deformities were observed in boys treated for delayed puberty onset (8). Further, aromatase-induced hyperandrogenemia may reduce high-density lipoprotein and increase hemoglobin levels (9). Treatment with growth hormone and letrozole has been used previously to improve the height of boys with idiopathic short stature. This case also showed vertebral changes, but it was in line with the typical manifestations of MS, and it had appeared before letrozole treatment. This is the first report of successful treatment using this regime in a patient with MS. Our results indicate that letrozole therapy is a safe and effective option in patients with MS and is free from any adverse side effects such as increased bone age. However, the long-term safety and efficacy of treatment with aromatase inhibitors in males is not well-established and should therefore be used with caution.

Increased testicular volume was observed throughout the course of this patient's treatment, a finding not previously reported. Earlier reports on sexual dysplasia associated with MS include precocious puberty, cryptorchidism, secondary amenorrhea, and delayed sexual development (10). We speculate that MS may cause dysfunction of the hypothalamic-pituitary-gonadal axis; however, the underlying mechanisms need to be further investigated.

In summary, we describe a case of MS in a child from mainland China. Following the wishes of the boy and his family, we treated him with growth hormone combined with letrozole and advised him to practice yoga. Following treatment, the boy's height increased, and he was able to squat better. Previous reports on MS focus on the clinical manifestations; however, we suggest using safe and appropriate individualized treatment considering the wishes of patients to improve their quality of life.

## Data Availability Statement

The raw data supporting the conclusions of this article will be made available by the authors, without undue reservation.

## Ethics Statement

Ethical review and approval was not required for the study on human participants in accordance with the local legislation and institutional requirements. Written informed consent to participate in this study was provided by the participants' legal guardian/next of kin. Written informed consent was obtained from the individuals, and minors' legal guardian/next of kin, for the publication of any potentially identifiable images or data included in this article.

## Author Contributions

XW and HW were attending physicians of the patient. HW performed the literature review, and wrote the first draft of the manuscript. YC and XW assisted in the patient's treatment. XW critically revised the paper. All authors contributed to the article and approved the submitted version.

## Conflict of Interest

The authors declare that the research was conducted in the absence of any commercial or financial relationships that could be construed as a potential conflict of interest.

## Publisher's Note

All claims expressed in this article are solely those of the authors and do not necessarily represent those of their affiliated organizations, or those of the publisher, the editors and the reviewers. Any product that may be evaluated in this article, or claim that may be made by its manufacturer, is not guaranteed or endorsed by the publisher.
